# Understanding fluoride adsorption from groundwater by alumina modified with alum using PHREEQC surface complexation model

**DOI:** 10.1038/s41598-023-38564-1

**Published:** 2023-07-29

**Authors:** Francis Adu-Boahene, Patrick Boakye, Frank Ofori Agyemang, Jolly Kanjua, Sampson Oduro‑Kwarteng

**Affiliations:** 1 Department of Civil Engineering (Regional Water and Environmental Sanitation Centre, Kumasi), Kwame Nkrumah University of Science and Technology, PMB, Kumasi, Ghana; 2 Department of Chemical Engineering, Kwame Nkrumah University of Science and Technology, PMB, Kumasi, Ghana; 3 Department of Materials Engineering, Kwame Nkrumah University of Science and Technology, PMB, Kumasi, Ghana; 4 Institute of Computation and Neuroscience, Apemso, Kumasi, Ghana

**Keywords:** Chemical engineering, Pollution remediation

## Abstract

Fluoride is recognized as a vital ion for human and animal growth because of the critical role it plays in preventing skeletal and dental problems. However, when it is ingested at a higher concentration it can cause demineralization of teeth and bones resulting in fluorosis, therefore, the production of high-adsorptive capacity material which is also cost-effective is necessary for the treatment of fluorides. In this study, aluminium foil is valorised into alumina nanoparticles. The as-prepared alumina was modified with alum in two different ratios of 1:0.5 and 1:1 (alumina to alum w/w%) and later used as adsorbents for the removal of fluoride from groundwater. The adsorbents were characterized by Fourier transform infrared spectroscopy, point of zero charge and X-ray diffraction. Different factors that influence the removal efficiency of fluorides such as pH, initial concentrations, contact time and adsorbent dosage were studied and optimized using a simulated fluoride solution. The optimum conditions obtained were used to test real groundwater. The static experiment conditions were used to calibrate a PHREEQC geochemical model which was later used to simulate the fluoride sorption onto the modified alumina at different conditions. PHREEQC was also coupled with parameter estimation software to determine equilibrium constants for the surface reactions between the fluoride species and the adsorbent in a way that the simulations accurately reflect the outcomes of laboratory experiments. Isotherm studies were carried out on the adsorbents. Both Langmuir and Freundlich's non-linear models fitted well for the equilibrium data. However, with a higher coefficient of regression and low chi-square test values, the adsorption process was more of chemisorption on a monolayer surface. Kinetic studies were also carried out by using the non-linear equations from the pseudo-first-order and pseudo-second-order models. The pseudo-second-order model fitted well for the equilibrium data. The mechanism for the fluoride ion adsorption was also studied by the intraparticle (IP) diffusion model and was found that IP was not the rate-determining factor, and therefore the most plausible mechanism for the sorption process was ion exchange or attraction of fluoride ions to the sorbent surface. The findings obtained from this research show that readily available aluminium waste could be valorised into a useful product that could be employed in the removal of fluoride from water samples, including groundwater, that may contain too much fluoride and pose a risk to the general public's health.

## Introduction

Fluoride is recognized as a vital element for human and animal growth because of the critical role it plays in preventing skeletal and dental problems. However, when it is ingested at a higher concentration it can cause the demineralization of teeth and bones resulting in fluorosis^1^. Fluoride is one of the crucial ions in addition to nitrate and arsenic which causes a wide range of health challenges via exposure. The maximum allowable concentration recommended in drinking water by the World Health Organization^2^ and the Ghana Standard Authority^3^ is 1.5 mg/L.

The associated health problems emanating from the ingestion of drinking water with higher concentrations of fluoride are on the rise in developing countries due to the lack of suitable water treatment facilities^4^. Natural sources of fluoride are interconnected with the different kinds of rocks and volcanic activities that manifest in those areas. Other contributing factors such as rock weathering, and mineralogy of watersheds and aquifers also account for the higher concentration of fluoride within a specific community^5^. Most people residing in these areas depend on groundwater for their water supply.

Due to the above-mentioned challenges associated with ingesting high concentrations of fluorides, there is the need to reduce the concentration to acceptable threshold or even below. Conventional defluoridation methods include; ion-exchange^6^, precipitation^7^, electrodialysis^8^, adsorption^9^, and reverse osmosis^10^. Adsorption has been shown in studies on the treatment of effluents containing fluorides to be a highly efficient and affordable method for removing fluorides from water^11^. The adsorption process is versatile in terms of design and operation, and it produces superior treated effluent in many cases^12^. Adsorbents can also be regenerated by a suitable desorption process because adsorption is sometimes reversible.

Several studies have investigated diverse sorbents for the defluoridation process. These include fly ash, activated and amorphous alumina, zirconium oxide, calcite, activated carbon from different biomass, clay minerals, agricultural wastes, rare earth oxide, charcoal, pumice and volcanic rock materials^13–15^. These materials are readily available in the environment but their adsorption capacity is normally not efficient, hence, better and more efficient adsorbents need to be developed^16^.

One of the essential constituents of municipal solid waste is aluminium which is obtained in terms of candy wrapping, cigarette, and aluminium foils^17^. One of the primary sources of aluminium waste that is problematic to recycle is the aluminium foil and therefore they are left with the option to either be buried or burned for disposal^18^. However, these wastes could be valorized into useful products such as alumina for the treatment of high fluoride concentrations from groundwater. Despite the fact that alumina is thought to be a good fluoride adsorbent, its efficacy could reach a moderate removal efficiency. Ghorai and Pant^19^ studied the defluoridation of water samples at an initial fluoride concentration of 12 mg/L using activated alumina and found that the removal efficiency was about 76% at a dosage of 16 g/L and a pH of 7.5.

Currently, automated calibration methods have attracted the interest of many scientists studying the adsorption of elements from aqueous solutions^20^. This is extremely helpful in situations where there is lack of experimental data for novel and non-conventional materials to generate precise predictions of sorption processes^21^.

In this research, the adsorption of fluoride from aqueous solution at various conditions onto alumina impregnated with alum was modelled and simulated using PHREEQC (which means pH, Redox, Equilibrium, and C programming language) geochemical modelling software coupled with parameter estimation software (PEST). The AlOH functional group present in the modified alumina served as the active sites used in the simulation. As far as the authors' knowledge is concerned, there has not been evidence of studies simulating the sorption of fluoride onto modified alumina by alum using computer models that would accurately predict the adsorption processes being successful before real process implementation. Previous studies have mainly focused on experimental design or field testing^16^. In this way, it could be possible to precisely predict how fluoride would react with the adsorbent under various conditions. Therefore, before carrying out the sorption process, one can utilize PHREEQC to predict the amount of the adsorbent to be employed based on feed water quality and the volume of the water to be treated.

The static experiment conditions were used to build the PHREEQC geochemical model. The calibrated model was used to investigate the effect of solution pH and adsorbent dosage on the removal of fluoride from an aqueous solution.

## Materials and method

### Materials

Virgin aluminium foil and Whatman filter paper 1 were obtained from DAMIMA Chemical Company Limited. Sodium carbonate, sodium hydroxide, and aluminium sulphate were obtained from Qualikems Fine Chemical Pvt Ltd. Sodium fluoride (97%), glacial acetic acid and sodium chloride and hydrochloric acid were purchased from Merck Company Ltd. Cyclohexylenediamine tetra acetic acid (CDTA) was also purchased from Hach Company Ltd.

### Alumina synthesis

The virgin aluminium foil was shredded into 10 × 10 mm size. Then, 5 g of the shredded foil was digested in 70 mL of concentrated hydrochloric acid (37%) under continuous stirring to obtain aluminium chloride solution based on Eq. (1).

1 $$2{\text{Al}}^{3 + } + 6{\text{HCl}} \to 2{\text{AlCl}}_{3} + 3{\text{H}}_{2}$$

The reaction is exothermic and is accompanied by heat and the release of a large amount of hydrogen gas as can be seen in Eq. (1) and is therefore recommended to be carried out in a fume chamber. The digested aluminium foil solution is diluted with an equal volume of distilled water (70 mL). The final solution is magnetically stirred at room temperature for 1 h to dissolve any unreacted foil using a magnetic stirrer. After the stirring, the solution is filtered three times under gravity using Whatman 1 filter paper to obtain a clear aluminium chloride solution. After, 10 g of sodium carbonate was weighed and dissolved in 50 mL of the aluminium chloride solution as shown in Eq. (2).

2 $$2{\text{AlCl}}_{3} + 3{\text{Na}}_{2} {\text{CO}}_{3} \to {\text{Al}}_{2} {\text{O}}_{3} + 3{\text{CO}}_{2} + 6{\text{NaCl }}$$

The by-product (sodium chloride) formed from the reaction was eliminated by washing the product with 500 mL distilled water several times until the supernatant pH dropped to 7. After the final wash, the product was placed in an evaporating dish and dried for 12 h at 105 °C. The dried sample was then crushed using mortar and pestle and sieved to 90 microns. The obtained particle size was then calcined at 550 °C for 4 h^22^ using a Nabertherm muffle furnace at a heating rate of 10 °C/min. The calcined sample was collected and kept in an airtight Ziploc bag and used for all necessary characterizations and adsorption experiments. This was thereafter called as-prepared alumina (A1).

### Alumina modifications

The surface of the as-prepared alumina (A1) was modified by using aluminium sulphate (alum) in a process as outlined by Waghmare et al.^23^ and Tripathy et al.^16^. This was done to increase the adsorption efficiency of the as-prepared alumina by impregnation with the aluminium sulphate which has a high fluoride adsorption capacity. The surface modification was obtained in a ratio of 1:0.5 and 1:1 (w/w alumina: alum) hereafter referred to as A2 and A3 respectively. The aluminium sulphate was dissolved in 100 mL of distilled water and the alumina was later added and stirred continuously for 1 h. Then the pH of this slurry was maintained in the range of 7–8 by adding NaOH and HCl. The alumina remained in contact with the alum for 3 h until equilibrium was obtained. After the contact time has elapsed, the slurry was filtered and the material obtained was washed with distilled water 3 times. Then the material was dried at 105 °C for 2 h in an oven.

### Material characterization

#### FTIR and XRD

**Figure 1:**
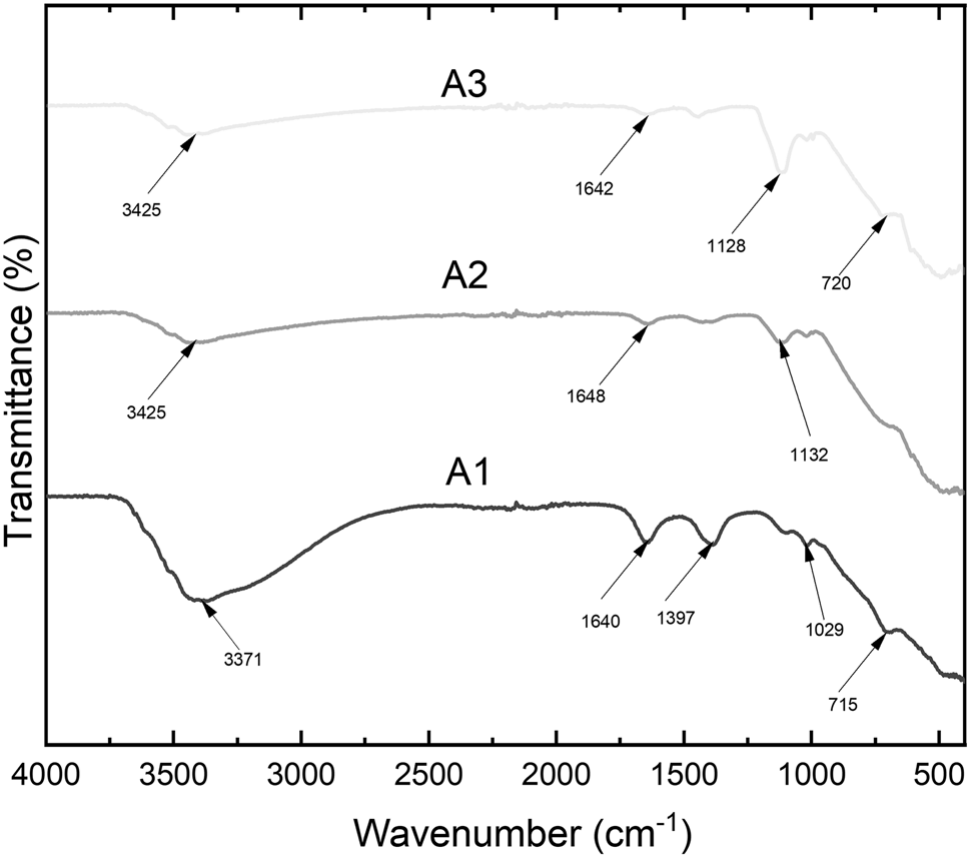
FTIR spectra of (A1) as-prepared alumina (A2) modified alumina with ratio of 1:0.5 and (A3) modified alumina with ratio of 1:1.

**Figure 2:**
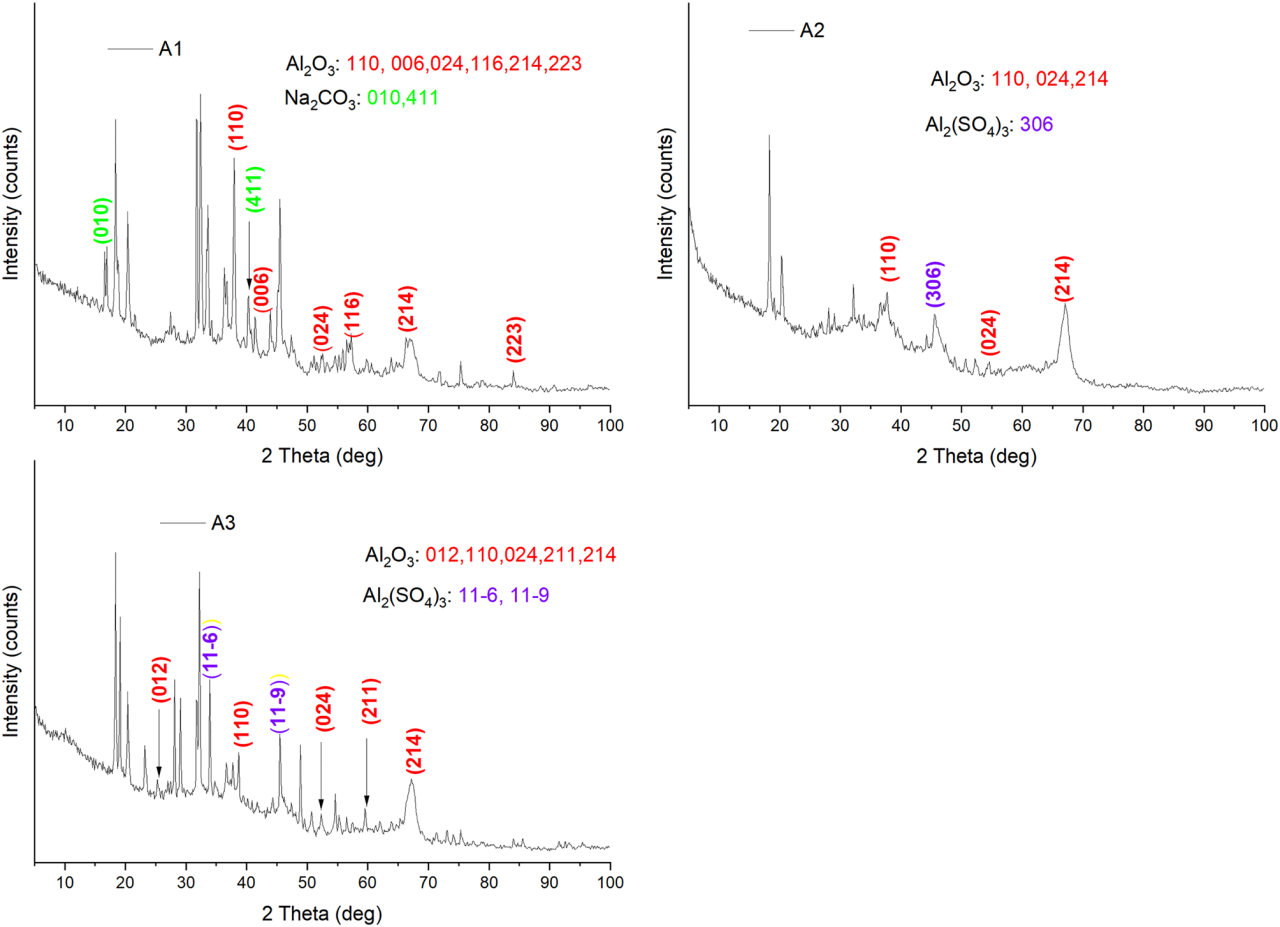
XRD patterns for (A1) as-prepared alumina (A2) modified alumina with ratio 1:0.5 and (A3) modified alumina with ratio 1:1.

The FTIR spectra were obtained by using Bruker–Vertex 60. The various surface functional groups present in the adsorbents that may aid in the adsorption process were identified using the FTIR analysis. This was observed within a wavelength of 4000 to 400 cm^-1^ (Fig. 1). The X-ray diffractometer (Panalytical Empyrean Series 2) was employed to study the structure of the adsorbents. Copper K alpha served as the radiation source and Nickel was used as the filter medium for the analysis. The K radiation was maintained at 1.54 Å and using a current and voltage of 40 mA and 45 kV respectively. The diffraction pattern is presented in Fig. 2.

#### Point of zero charge

The point of zero charge was determined using the solid addition method as described by Todorovi & Milonji^24^ with little modification. Exactly 0.1 M potassium nitrate solution was prepared and used as an inert electrolyte. Then, 25 mL of the potassium nitrate solution at different pHs ranging from 3–12 was obtained by adjusting the initial pH by 0.1 M potassium hydroxide and 0.1 M nitric acid solutions. The measured volumes were transferred in the different glass vials and 100 g of the adsorbents were added. The solution was shaken for 24 h until equilibrium was obtained at 30 °C and a rotational speed of 150 rpm. After equilibrium has been achieved, the solution was filtered and the final pHs were measured. The difference between the initial and the final pH obtained was as:

3 $$\Delta pH = pH_{i} - pH_{f}$$

A graph of $\Delta pH$ against ${pH}_{i}$ is obtained and the point that crosses the zero axis is recorded as the point of zero charge of the particular adsorbent.

### Adsorption experiment

Exactly 100 mg/L of fluoride standard solution was prepared by dissolving 227.89 mg of 97% pure sodium fluoride in 1000 mL distilled water. The solution was stirred continuously to dissolve all the sodium fluoride in the distilled water. Serial dilution was done to prepare other concentrations for the static adsorption studies. Total ionic strength adjustment buffer (TISAB II) which plays an essential role in the determination of F^-^ ions by adjusting the ionic strength, buffer of the pH and break up metal fluoride complexes was also prepared by following the protocol outline by^25^. Then, 1 L beaker was filled with 500 mL distilled water. After, 58 g of sodium chloride, 4 g of CDTA were weighed and 57 mL of glacial acetic was added to the 500 mL of distilled water. The mixture was stirred continuously to form a homogenous solution. To obtain a final pH of 5.5, the solution was adjusted by a 5 M sodium hydroxide solution. The solution was transferred into a 1000 mL conical flask and was topped up to the 1000 mL mark using distilled water. Static adsorption tests were performed with the different types of adsorbents. These were the as-prepared alumina (A1), modified alumina with alum at different ratios. Alumina-alum; A2 and A3 at 1:0.5 and 1:1 ratio respectively. Specific fluoride concentrations were prepared via dilutions of a previously standard stock solution (100 mg/L). Afterwards, 10 mg of each adsorbent was measured into 100 mL of a borosilicate glass vial and filled with 10 mL of 5 mg/L fluoride solution for contact time studies (5–180 min) at initial pH 6.5. The mixture was kept at 30 °C under an orbital shaker (DF-LI-00080 SS1 LAB) at 150 rpm until equilibrium was attained. The influence of initial fluoride concentration was also studied at 1, 5, 10, 15 and 30 mg/L after obtaining the optimum equilibrium time. The effect of initial solution pH (initial conc. 5 mg/L, t = 60 min, speed = 150 rpm) on the adsorption of fluoride by the adsorbents was investigated from 3 to 12. The initial pH of the fluoride solution was adjusted using 0.1 M HCl and 0.1 M NaOH solution. The effect of adsorbent dosage was carried out in different adsorbent masses at 10, 30, 50, 70 and 90 mg at the optimum conditions obtained in the previous experiments. The sample solution was filtered using Whatman paper 1 and 5 mL of the filtrate was measured and an equal volume of the TISAB II was added before the reading was taken.

The amount of fluoride in the solution before and after adsorption was measured with HACH HQ40d portable fluoride meter connected to an IntelliCAL fluoride ion-selective electrode probe (ISEF121). The amount of fluorides adsorbed by the adsorbents and the percentage removal were calculated using Eqs. (4) and (5) respectively;

4 $$q_{e} = \frac{{\left( {C_{o} - C_{e} } \right)xV}}{m}$$

5 $$\% {{Re}} moval = \frac{{\left( {C_{o} - C_{e} } \right)}}{{C_{o} }} x 100\%$$

where ${q}_{e}$(mg/g) is the equilibrium adsorptive capacity. Co (mg/L) is the fluoride initial concentration and Ce (mg/L) is the fluoride equilibrium concentration in the solution. V (L) is the working volume of the fluoride solution and m (g) is the adsorbent dosage used. The adsorption process was repeated in duplicate and the mean and standard deviation of the data were reported.

### PHREEQC model development

The PHREEQC geochemical modelling code version 3.7.3–15,968^26^ was used to model and simulate the adsorption of fluoride onto the modified alumina at various conditions. PHREEQC can be used to determine the concentration of adsorbate in an aqueous solution, uptake, and percent removal of an adsorbent. When all the necessary information is included in the input script, the interaction of the adsorbate and the adsorbent can be precisely determined. The input script used in the simulation is given in Table 1. The “Alum_al” denotes the AlOH functional group on the modified alumina. PHREEQC also allows the user to specify other parameters such as the number of moles surface sites (mol), specific surface area (m^2^/g), and dosage (g) of the adsorbent. These three parameters are necessary for defining the properties of the adsorbent. Other parameters such as temperature, feed water quality, the volume of feed, etc. are used to define the solution used in the simulation. All conditions used in the static adsorption process were used to calibrate the model. The built-in WATEQ4F database was chosen because it has all the relevant analytes and the laboratory settings that serve as a good representation of field parameters.

**Table 1:**
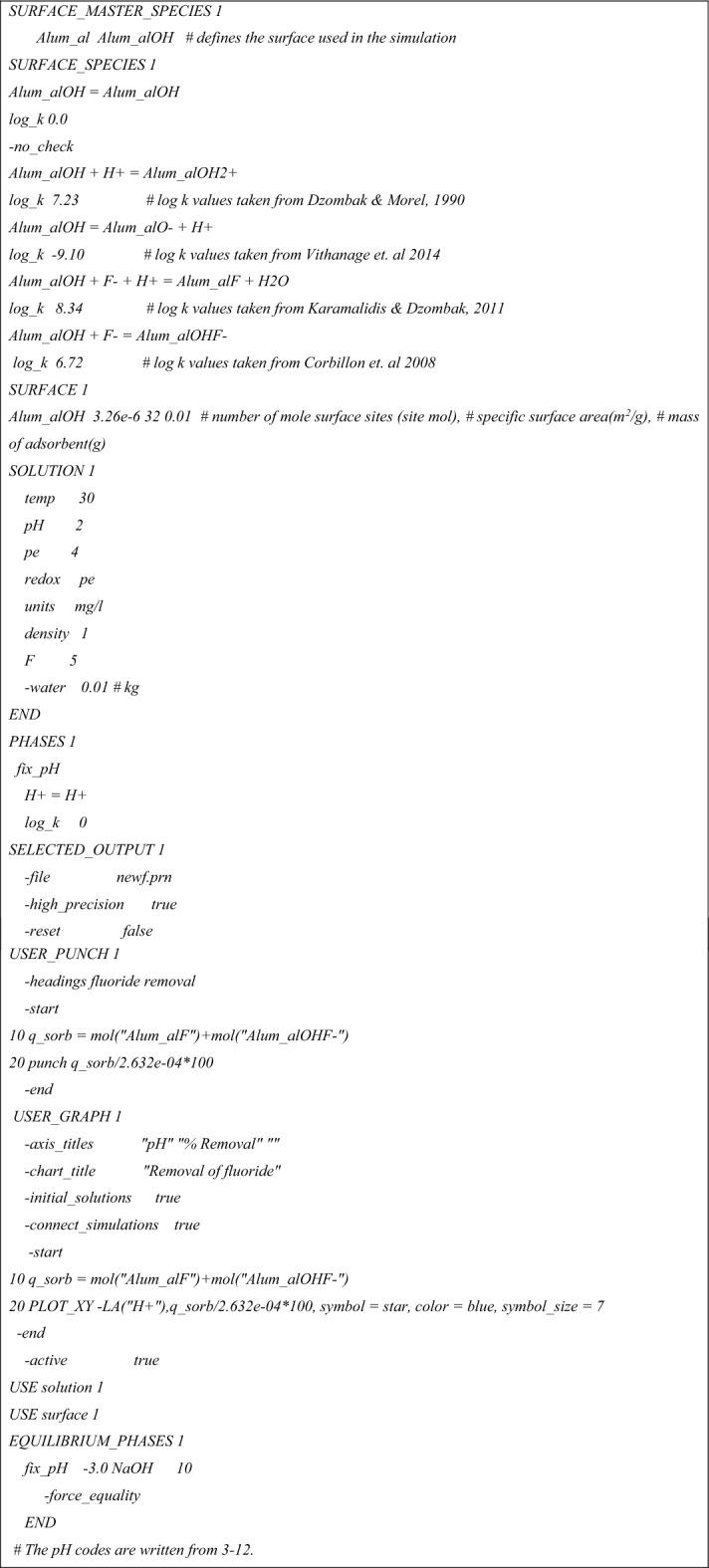
Input script for PHREEQC model development.

### Parameter estimation (PEST)

Similar to the methods covered in^21,27^, model-independent parameter estimation (PEST) was utilized in this research to precisely calculate the equilibrium constants of the reactions taking place between the adsorbent surface sites and the fluoride species. The initial log k values used in Table 1 were obtained from multiple sources in literature^28,29^. In PEST, equilibrium constants were calibrated by comparing model outputs to measurements of the system state.

### Sampling of real groundwater

The water was sampled using a 100 mL clean container and filtered through a 0.45 mm membrane into a clean 350 mL already prepared plastic bottle which was later capped. Each sample container was labelled correctly with a unique code that included the identification number, date, time, and sample designation and later refrigerated with the prime aim of preserving them at 4 °C on the field before being transported to the laboratory. In addition, the GPS coordinates were recorded via Garmin 62Stc global positioning system (GPS) device at the time these samples were collected. Because fluoride detection from water samples does not require any special handling, other than storing at the proper condition for 28 days, the collected samples were delivered to the lab for further analyses.

### Adsorption isotherms

The equilibrium isotherm data were analyzed using the non-linear forms of the Langmuir and Freundlich isotherm models. The non-linear regression coefficients (R^2^) and chi-square test results were used to assess the models. The non-linear expression of the Langmuir isotherm model is given as:

6 $$q_{e = } \frac{{q_{m} K_{L} C_{e} }}{{1 + K_{L} C_{e} }}$$

where; ${q}_{e}$(mg/g) is the equilibrium adsorptive capacity, Ce (mg/L) is the equilibrium adsorbate concentration in the bulk solution, q_m_ (mg/g) is the Langmuir maximum adsorptive capacity and K_L_ (L/mg) is the Langmuir constant associated with the energy of the adsorption process.

The non-linear Freundlich adsorption isotherm model is also given as;

7 $$q_{e} = K_{F} C_{e}^{\frac{1}{n}}$$

where K_F_ (mg/g)(L/mg)^1/n^ is the Freundlich coefficient which is linked to the adsorption capacity of the sorbent and 1/n is a dimensionless factor called Freundlich intensity which defines the intensity of the adsorption process or heterogeneity of the surface of the sorbent. The values of 1/n range from 0 to 1. When 1/n > 1 it shows that the adsorption process is unfavourable; 0 < 1/n < 1 means favourable adsorption process. 1/n = 0 and 1/n = 1 mean irreversible and linear adsorption processes respectively. The 1/n values are used to envisage the shape of the isotherms^30^. A better fit of adsorption equilibrium data to this model indicates that the sorption of the adsorbates involving multilayer adsorption on the surface of the sorbent is heterogeneous.

### Adsorption kinetics

Kinetic studies in static adsorption provide information about optimum conditions, sorption mechanism, and possible rate-controlling step. Adsorption kinetics measure adsorbate uptake with respect to time at constant pressure or concentration, and it is used to regulate adsorbate diffusion in the pores of the material. In this research, the kinetics data obtained from fluoride adsorption using the different adsorbents were subjected to the non-linear forms of the pseudo-first order and pseudo-second order, as well as the intraparticle diffusion models.

The non-linear form of the Lagergren pseudo-first-order (PFO) kinetics model is expressed as:

8 $$q_{t} = q_{e} \left( {1 - e^{{ - K_{1} t}} } \right)$$

where q_t_ (mg/g) is the adsorptive capacity obtained at a predetermined time, t, (min) and q_e_ (mg/g) is the equilibrium adsorptive capacity. K_1_ (1/min) is the pseudo-first-order rate constant. Since K_1_ is a time scaling factor, its value indicates how sooner equilibrium is reached during the sorption process. High values of K_1_ helps the system to reach equilibrium faster.

The non-linear form of the pseudo-second-order (PSO) kinetics model is also expressed as:

9 $$q_{t} = \frac{{q_{e}^{2} K_{2} t}}{{1 + q_{e} K_{2} t}}$$

where K_2_ (g/mg.min) is the pseudo-second-order rate constant.

The intra-particle diffusion rate model is given by;

10 $$q_{t} = K_{p} \sqrt {t } + C$$

where K_p_ is a rate constant mg/g.min^0.5^, and C is boundary layer thickness. The values obtained from C determine the effect of the boundary layer. A higher C value implies a higher boundary layer effect implying pore diffusion is not the sole rate-controlling mechanism describing the dynamics of the adsorption process.

### Statistical analysis

The correlation between the experimental and modelled data was determine using SigmaPlot v14.0 software by employing the Mann–Whitney paired-sample and Student unpaired t-test. The data was first subjected to the normality test to identify whether it is parametric or non-parametric (*p* = 0.05 at a confidence level of 95%).

## Results and discussions

### Characterization of adsorbents

#### FTIR

The Fourier Transform Infrared spectra were utilized to analyse the functional groups of all the adsorbents that may aid in the adsorption process. Figure 1 shows the FTIR spectra of the three adsorbents.

The broad peak observed at 3371 cm^−1^ in the as-prepared alumina (A1) is attributed to the Al–OH stretching of the -OH group^31^. The peak is shifted to 3425 cm^-1^ in the A2 and A3 as the surface of the A1 is modified by alum and this narrowed the spectra. The peak which appeared at 1640 cm^-1^ in A1 is probably due to the O–H distortion of water which is absorbed by the high surface area of the alumina^32^. This peak was shifted to 1648 cm^-1^ and 1642 cm^-1^ in A2 and A3 respectively due to the bending vibration of SO_4_ ions from the alum to the pristine material. The peaks at 1128 cm^-1^ and 1132 cm^-1^ in A3 and A2 respectively signify the triply degenerative vibrational mode of sulphate ion because of the surface modification of the aluminium sulphate^33^. The peak appearing at 1029 cm^-1^ in A1 is due to the symmetrical bending vibration of Al–O–H group^34^. The band observed at 720 cm^-1^ and 715 cm^-1^ A3 and A1 respectively corresponds to the stretching vibrations of the aluminium-oxygen (Al-O) bonds in the crystal lattice structure of the material.

#### XRD

XRD pattern was used to determine the crystalline phases of the adsorbents. The pattern provided in Fig. 2, shows the pristine material (A1), and the modified adsorbents A2 and A3 based on the different aluminium sulphate ratios.

The conspicuous reflections observed in all the adsorbents revealed that the main crystalline phases present in the adsorbents were aluminium oxides according to the ICDD reference number 00–010-0173. The dominant peaks for A1 were observed at 2θ = 37.79, 41.63, 52.45, 57.33, 66.28, 84.08 °C which are indexed as (110), (006), (024), (116), (214) and (223) respectively. A similar pattern was also reported in previous studies^35^. It was also evident that traces of sodium carbonate that reacted with the aluminium chloride solution to produce the alumina peaks were observed at 2θ = 16.83 (010) and 40.27 (411) °C (JCPDS-c08-0448)^36^. The traces were seen as not all of the sodium carbonate completely reacted to produce the alumina. The crystalline phases in A2 and A3 were recorded at 2θ = 37.71 (110), 52.21 (024), and 67.13 (214) °C for A2 and 2θ = 25.44 (012), 37.76 (110), 52.33 (024), 59.56 (211) and 67.24 (214) for A3. Similar patterns were also obtained by Ghulam et al*.*^22^ and Gu et al*.*^37^. Peaks for the aluminium sulphate used for the modification were identified in A2 at 2θ = 46.77 (306) according to the ICSD code of 073,249. Two more peaks were identified in A3 at 2θ = 33.94 ($11\overline{6}$) and 44.26 ($11\overline{9}$) °C^38^.

#### Point of zero charge

The point of zero charge defines the condition of the surface of a dispersed solid phase at a solid-electrolyte solution interface. This is the pH at which the positive and the negative surface concentrations are in equilibrium. The surface of the material (adsorbent) becomes positive when the pH of the solution is less than the point of zero charge (pH < pHpzc) and negative when the pH is greater than the point of zero charge (pH > pHpzc).

**Figure 3:**
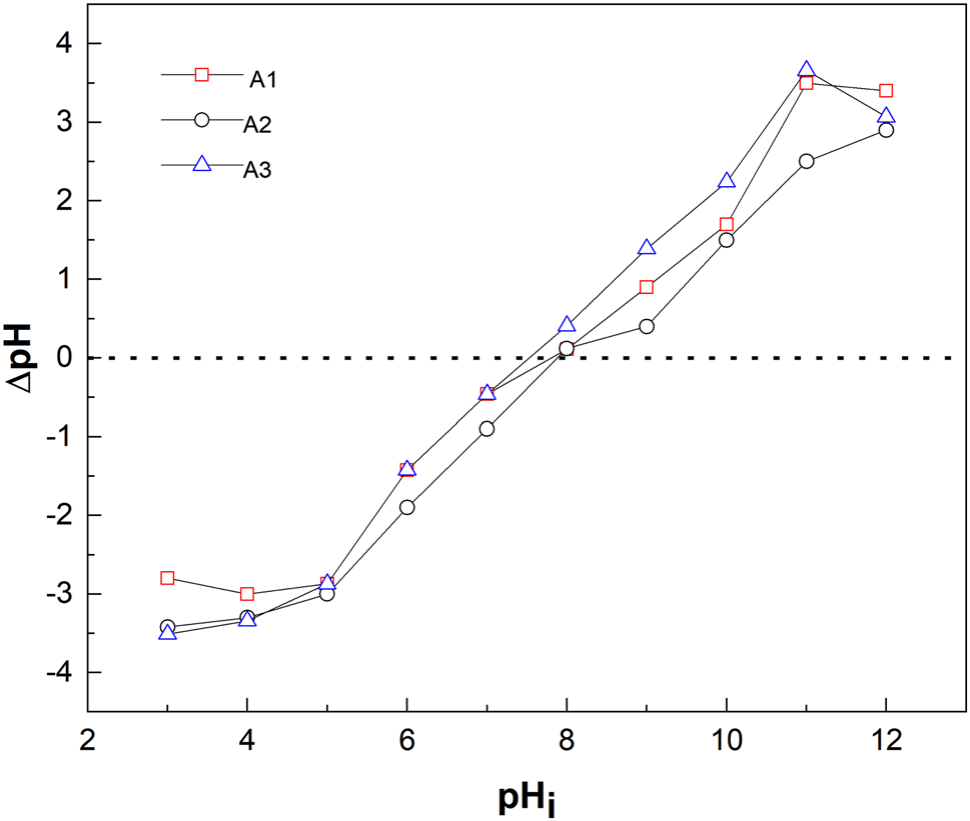
Point of zero charge of (A1) as-prepared alumina (A2) modified alumina with ratio 1:0.5 and (A3) modified alumina with ratio 1:1.

For this study, the point of zero charge was obtained by plotting the change in pH ($\Delta pH)$ against initial pH (pH_i_) and the line that crosses the zero axis was recorded. The result for the various adsorbents is depicted in Fig. 3. The pHpzc for A1 and A2 was obtained at pH value of 8.0 and that of A3 at 7.5. The values obtained are consistent with the work of Kosma et al.^39^.

### Fluoride aqueous speciation

Fluoride aqueous speciation was calculated for a solution with a total fluoride of 5 mg/L. The speciation was computed by using PHREEQC interactive geochemical modelling code version 3.7.3–15,968^26^ with WATEQ4F thermodynamic database.

**Figure 4:**
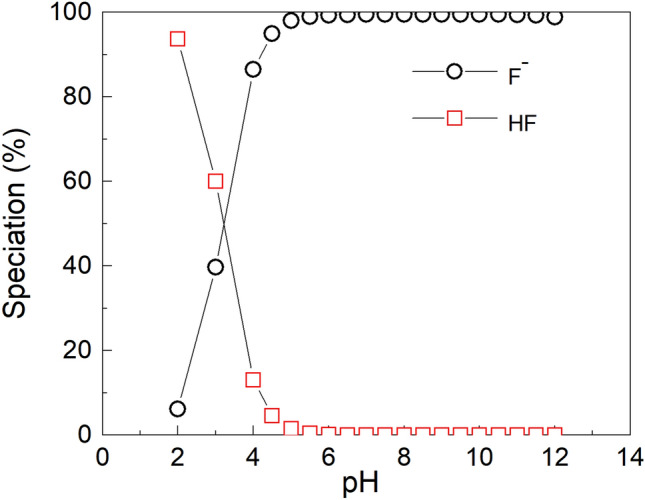
Fluoride aqueous speciation in 5 mg/L, and 30 °C calculated with PHREEQC geochemical codes using WATEQ4F database.

Fluoride speciation is presented in Fig. 4. It is evident that F^-^ is completely protonated in the acidic pH range up to 3. The protonated neutral species of HF will not be adsorbed in the acidic range. About 50% of F^-^ at pH 4 is protonated and above a pH of 5, F^-^ is completely deprotonated and will exist as free F ions. It is therefore expected that maximum F^-^ adsorption will occur at pH > 5.

### Influence of contact time on adsorptive capacity

**Figure 5:**
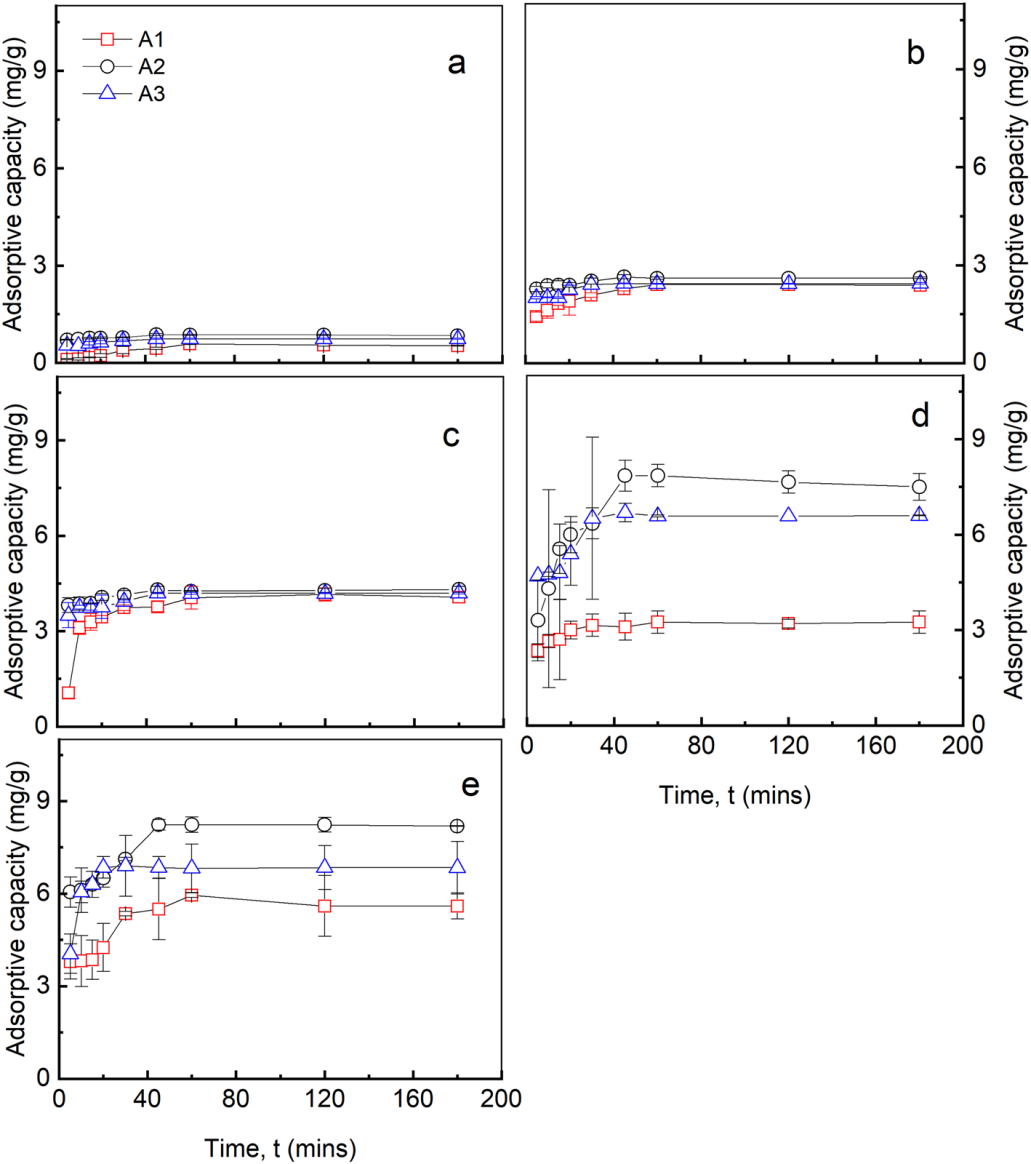
Influence of contact time on adsorptive capacity at initial fluoride concentration of (**a**) 1 mg/L (**b**) 5 mg/L (**c**) 10 mg/L (**d**) 15 mg/L (**e**) 30 mg/L, pH 6.5, dosage 10 mg, temp. 30 °C, working volume 10 mL, rotational speed of 150 rpm for A1 (as-prepared alumina), A2 (modified alumina with alum ratio 1:0.5), (A3) modified alumina with alum ratio 1:1. The error bars represent the standard deviations from the duplicate experiments (NB: some of the error bars are not visible because they are within the size of the marker).

The influence of contact time on fluoride ion adsorption by the adsorbents; as-prepared alumina (A1), modified alumina with alum at the ratios 1:0.5 and 1:1 as A2 and A3 respectively at initial fluoride concentrations 1, 5, 10, 15 and 30 mg/L were studied and shown in Fig. 5. During the adsorption process, the contact time was varied from 5 to 180 min at a fixed initial pH of 6.5, a working volume of 10 mL, mass of adsorbent of 10 mg, and a rotational speed of 150 rpm.

It could be seen from all the plots that, the adsorptive capacities increase as time increases until equilibrium was reached. Moreover, the adsorptive capacities also increased as the initial concentrations increased. For A1, an equilibrium contact time of one hour was observed with maximum adsorptive capacities of 5.95 mg/g at an initial fluoride concentration of 30 mg/L. Unlike A1, an equilibrium contact time of 45 min was observed in A2 and A3 with maximum adsorptive capacities of 8.23 mg/g and 6.85 mg/g respectively. It was also observed that the maximum fluoride uptake by all the adsorbents increased with an increase in initial fluoride concentration from 1 to 30 mg/L. These findings were obtained due to the high driving force obtained from the concentration gradient as a result of increase in initial fluoride concentration (C_0_).

### Influence of initial concentration

**Figure 6:**
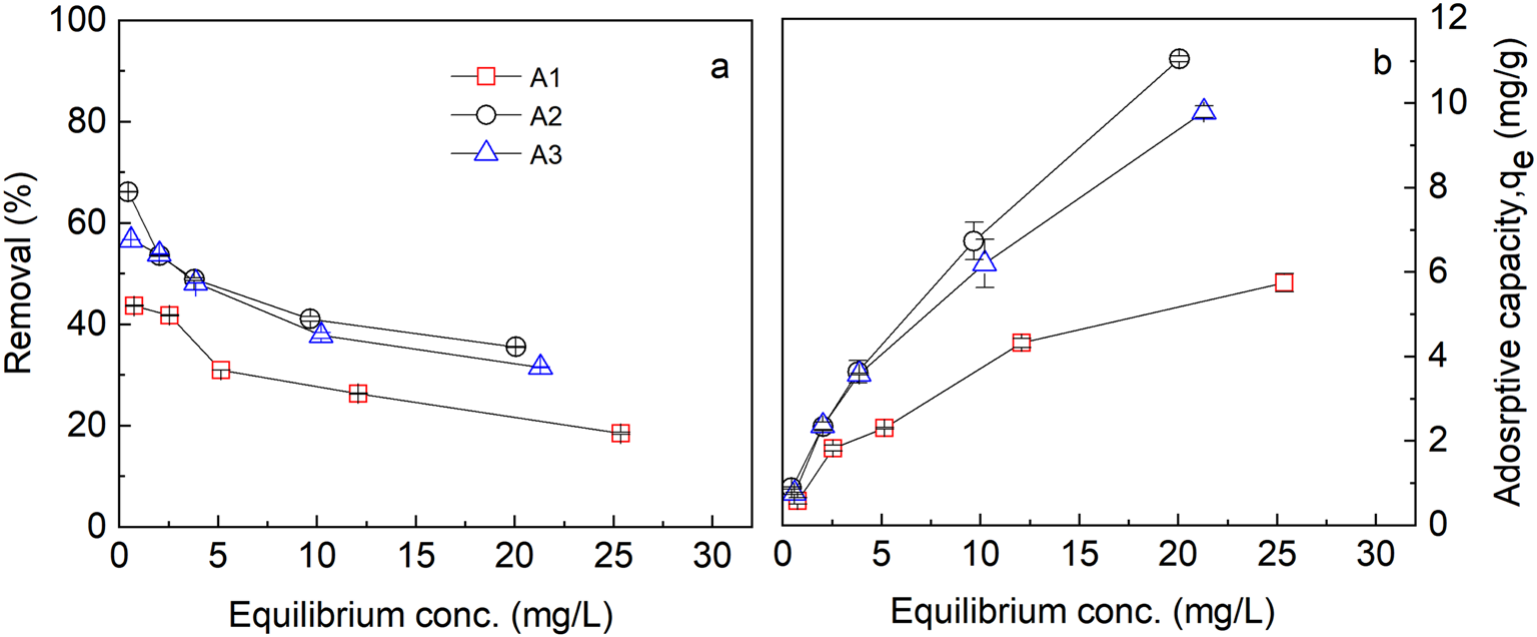
Influence of equilibrium concentration on (**a**) Percent removal (**b**) Uptake at initial fluoride concentration of 1–30 mg/L, pH 6.5, temp. 30 °C, time 1 h, working volume 10 mL, rotational speed of 150 rpm for A1 (as-prepared alumina), A2 (modified alumina with alum ratio 1:0.5), A3 (modified alumina with alum ratio 1:1). The error bars represent the standard deviations from the duplicate experiments (NB: some of the error bars are not visible because they are within the size of the marker).

The influence of initial concentration on the maximum adsorption uptake was studied by varying the initial concentrations from 1, 5, 10, 15, and 30 mg/L of a fluoride solution. The equilibrium contact time obtained from the previous experiment was used thus 1 h, an adsorbent dosage of 10 mg, temperature of 30 °C, initial pH solution of 6.5, a rotational speed of 150 rpm and a working volume of 10 mL. The maximum adsorptive capacities, q_e_ of the various adsorbents were plotted against the equilibrium concentrations and the result obtained is depicted in Fig. 6.

As it is depicted in Fig. 6, an increase in initial fluoride concentrations increased the maximum adsorptive capacities of all the adsorbents. The maximum adsorption capacity (q_max_) of fluoride onto the adsorbents was in the order A2 (11.03 mg/g) > A3 (9.8 mg/g) > A1 (5.75 mg/g). On the contrary, a different pattern was observed in the fluoride percentage removal from an initial concentration of 1 mg/L to 30 mg/L. This is true because, at higher concentrations, the active sites on the adsorbents become saturated owing to the existence of more adsorbates than the adsorption capacity of the adsorbents. The higher ratio of the adsorbates at constant adsorbent dosage over the readily available active sites with increasing initial adsorbate concentrations saturate the surfaces which reduces the sorption capacity hence the reduction in percent removal^40^. At low adsorbate concentrations, there are more readily available active sites on the adsorbent than the adsorbate and hence most of the adsorbates interact with these active sites during the sorption process. The percent removal increases until equilibrium is reached. Shimelis et al.^41^, Gomoro et al.^42^ and Wambu et al.^43^ reported a similar trend in their adsorption experiment, pointing out that as the initial concentrations of the adsorbate were increased, the percentage removal of fluoride by the adsorbent decreased.

### Effect of pH on adsorptive capacity

**Figure 7:**
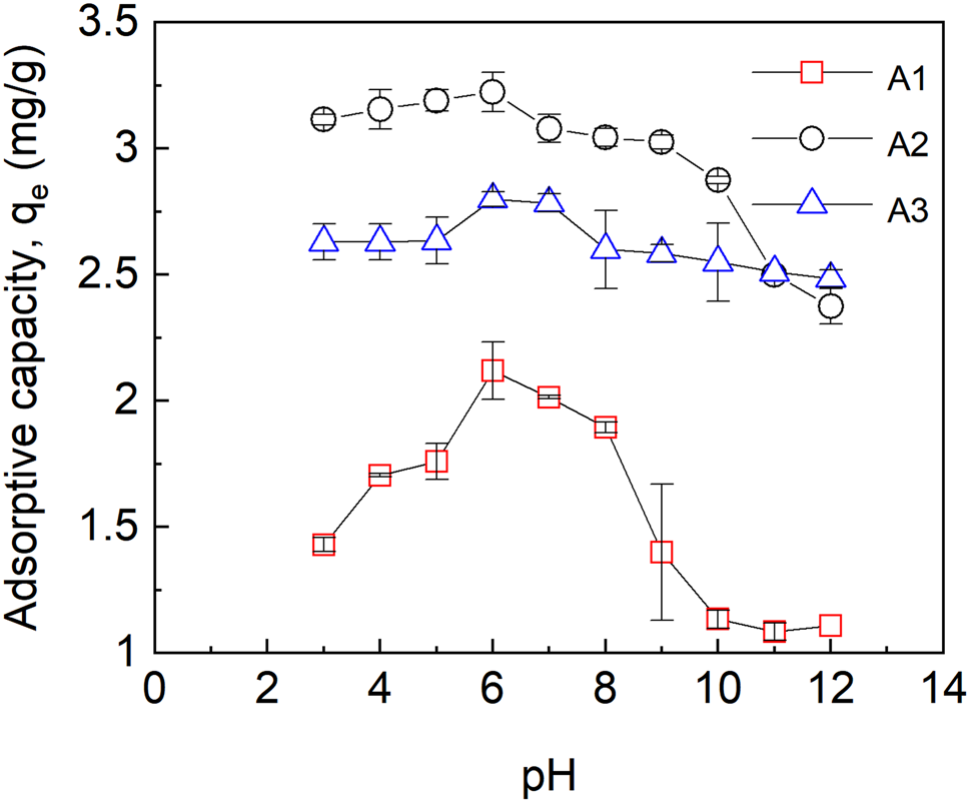
Effect of pH on adsorptive capacities at initial fluoride concentration of 5 mg/L, dosage 10 mg, temp. 30 °C, time 1 h, working volume 10 mL, rotational speed of 150 rpm for A1 (as-prepared alumina), A2 (modified alumina with alum ratio 1:0.5) and (A3) modified alumina with alum ratio 1:1. The error bars represent the standard deviations from the duplicate experiments (NB: some of the error bars are not visible because they are within the size of the marker).

The pH of a solution is a major parameter that affects the chemical species of a solute and the surface properties of adsorbents such as surface charges^44^. To ascertain the optimal pH suitable for fluoride sorption by the adsorbents (with an initial fluoride concentration of 5 mg/L, contact time;60 min, speed; 150 rpm, working volume;10 mL) studies were done over a pH range of 3–12. As is depicted in Fig. 7 the maximum adsorptive capacity of all the adsorbents depends on pH.

The adsorptive capacity increased as pH was increased until a maximum value was reached at pH 6.0, and a further increase in pH above 6.0 decreased the adsorptive capacity. The increase in the adsorptive capacities of A2 and A3 until equilibrium was seen to be gradual as compared to A1.

The point of zero charge (pHpzc) for A1 and A2 was found to be 8.0 and that of A3 was 7.5. At pH < pHpzc, the surface of the adsorbent was characterised by H^+^ ions and there was an electrostatic force of attraction between the adsorbate (F^-^) and the H^+^ ion, hence, the maximum adsorption at 6.0. At pH > pHpzc, the surface of the sorbent became negatively charged and was characterized by the presence of OH^-^ ions. Beyond the pHpzc of the sorbents, the adsorptive capacity decreased because of the electrostatic repulsion between the F^-^ ions and the OH^-^ ion. The formation of HF, which reduced the coulombic attraction between fluoride and the adsorbent surface, is thought to be responsible for the low fluoride removal capacity at acidic pH as shown in Fig. 4. Tabi et al.^45^ studied the removal of fluoride from simulated water using zeolite modified with alum and obtained a maximum percent removal of about 98 at a pH of 6. In a defluoridation process by Zhao et al.^46^ using Fe3O4@Al (OH)_3_ magnetic nanoparticles, maximum adsorption of fluoride was achieved in a pH range of 5 to 7.

### The effect of pH on the adsorption of fluoride and PHREEQC model calibration

The PHREEQC geochemical model codes were run to ascertain the effect of pH on fluoride sorption by the modified alumina (A2). A2 was chosen as the best adsorbent for the simulation based on results from the previous experiments. The equilibrium constants for the chemical reactions between the fluoride species and the adsorbent were adapted from multiple sources in the literature as given in Table 2.

**Table 2:** Surface reactions and parameters used in the simulation.

No	Reactions	Log k	References
1	Alum_alOH + H^+^ = Alum_alOH_2_^+^	7.23	^28,29^
2	Alum_alOH = Alum_alO^-^ + H^+^	− 9.10	^47^
3	Alum_alOH + F^−^ + H^+^ = Alum_alF + H_2_O	8.34	^29^
4	Alum_alOH + F^−^ = Alum_alOHF^−^	6.72	^48^

**Figure 8:**
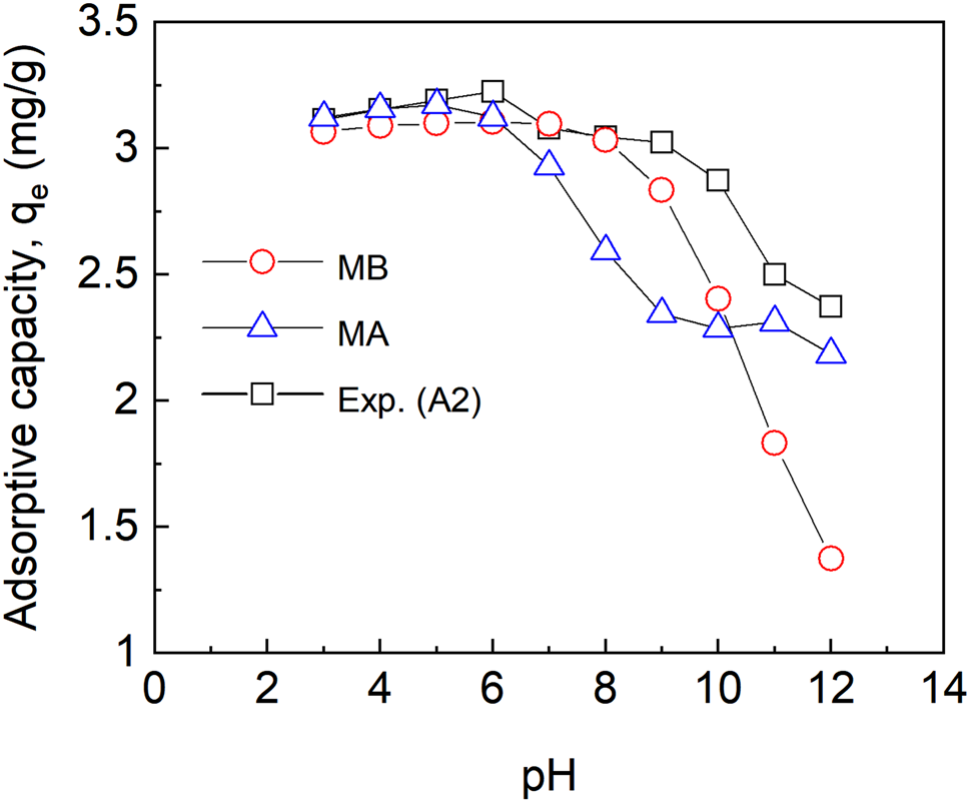
Influence of pH on adsorptive capacity at initial fluoride concentration of 5 mg/L, temp. 30 °C, working volume 10 mL for Exp. A2 (Experimental data), MB (modelled data before optimization) and MA (optimization after optimization).

The conditions used in the static adsorption experiments were used to calibrate the PHREEQC model using the effect of pH (3–12), initial fluoride concentration of 5 mg/L, an adsorbent dosage of 10 mg, and a working volume of 10 mL. The number of moles of surface site, specific surface area, and mass of the adsorbent used are needed to define the properties of the adsorbent in the simulation. The number of moles of surface sites was calculated using the equation given by Karamalidis & Dzombak^29^ by taking 7.5% of the specific surface area. A site density of 8 sites/nm^2^ and a specific surface area of 32 m^2^/g used in the calculation were taken from Karamalidis & Dzombak^29^. As it could be inferred from Fig. 8, the modelled data did not coincide with the experimental data. This could be attributed to the equilibrium constants employed in the simulation and therefore the model could not be used to simulate the fluoride sorption process. To solve this problem, the PHREEQC codes were coupled with parameter estimation software such as PEST to precisely estimate the equilibrium constants from the experimental data. The PHREEQC model´s input and output files are used to interact with PEST in the parameter estimation. To run the PEST optimization process, three files are needed. These include the PEST control file, instruction file, and template file. The parameters to be estimated (equilibrium constants) are detailed in the template file (Sup F1-Template file) which is a facsimile of the PHREEQC input file. The instruction file (Sup F2-Instruction file) is used by PEST to read the PHREEQC output file to make a comparison with the experimental data. The parameters that need to be adjusted to obtain a best fit between the experimental and model data are contained in the PEST control file (Sup F3-Control file). These parameters include; the algorithm used for the PEST optimization, the experimental data that will be used to calibrate the model output, initial and boundary values of model parameters. The Shuffled Complex Evolution Algorithm (sceua_p) in the PEST directory was used for the parameter optimization. The algorithm interacts with the PHREEQC model via the model's input and output files and estimates the equilibrium constants by carrying out several iterations until both the model´s output and the measured (laboratory observations) data are close as possible to each other.

The new optimized equilibrium constants as given in Table 3 were then used to calibrate the model in Table 1 and the result is depicted in Fig. 8. The Mann–Whitney paired-sample and Student unpaired t-test at a significant level of 0.05 were also carried out to determine the correlation between the experimental and modelled data.

**Table 3:** Optimized equilibrium constants values for the surface reactions.

No	Reactions	Optimized log k
1	Alum_alH + H^+^ = Alum_alOH_2_^+^	4.00
2	Alum_alOH = Alum_alO^-^ + H^+^	− 8.59
3	Alum_alOH + F^-^ + H^+^ = Alum_alF + H_2_O	5.84
4	Alum_alOH + F^-^ = Alum_alOHF^-^	7.48

It could be inferred from Fig. 8 that, the adsorptive capacities for the experimental data (A2) are higher than the modelled data. However, the Mann–Whitney paired-sample t-test revealed there was no statistical significance between the two data (*p* = 0.307 at a confidence level of 95%). The results indicate that PHREEQC coupled with PEST could be a powerful tool for estimating adsorption parameters. The new optimized equilibrium constants were used to simulate the influence of adsorbent dosage on the fluoride sorption process.

### Influence of adsorbent dosage

The number of active sites present on an adsorbent plays a critical role in the sorption process. The influence of an adsorbent dosage was investigated by changing the mass from 10, 30, 50, 70 to 90 mg. The other parameters such as contact time, pH, shaking speed and initial concentration were kept at their optimum conditions from the previous experiments. The results obtained were compared with the simulation from the new optimized equilibrium constants.

**Figure 9:**
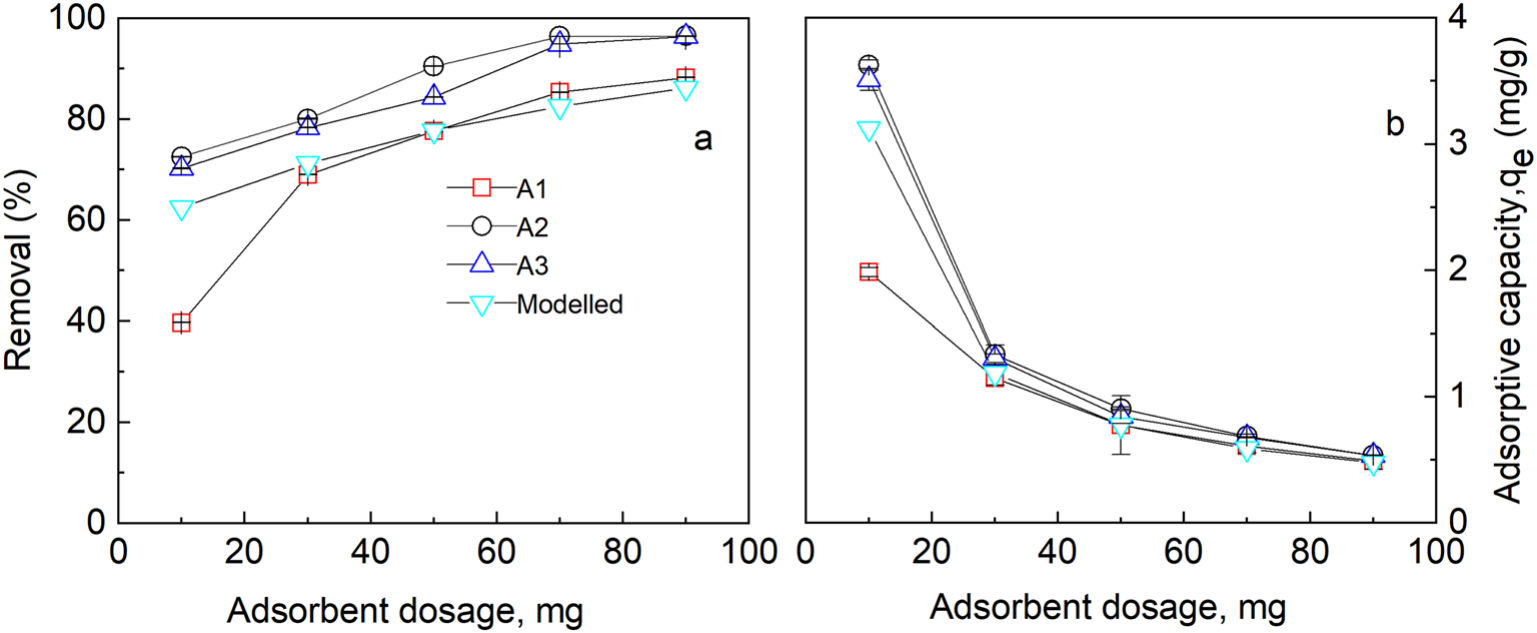
Influence of adsorbent dosage on (**a**) Percent removal (**b**) Uptake at initial fluoride concentration of 5 mg/L, pH 6, temp. 30 °C, time 1 h, working volume 10 mL, rotational speed of 150 rpm for A1 (as-prepared alumina), A2 (modified alumina with alum ratio 1:0.5), (A3) modified alumina with alum ratio 1:1 and PHREEQC model calculated with WATEQ4F database. The error bars represent the standard deviations from the duplicate experiments (NB: some of the error bars are not visible because they are within the size of the marker).

The removal efficiency was studied and the results are presented in Fig. 9. The figure reveals there was an increase in the percentage removal of fluoride as the dosage increased from 10 to 90 mg. This is attributed to the increase in the surface area and the number of active sites for a fixed number of adsorbates. Shimelis et al.^41^ observed a similar trend in defluoridation of aqueous fluoride solution by aluminium oxide. It could be observed in A2 and A3 that there was no considerable increase in the percent removal after a specified dosage of 70 mg with a removal efficiency of about 96% at that specified conditions. This might be explained by the existence of more excess adsorption sites than adsorbates, assuming that the number of adsorptions per unit mass of adsorbents remains constant. The Student unpaired t-test carried out between the experimental data (A2) and the modelled data reveals that there was no statistically significant difference between the experimental data (A2) and the modelled data (*p* = 0.373 at a confidence level of 95%). This suggests that the newly optimized parameters were able to simulate the adsorption of fluoride on the modified alumina. In the case of A1, there was an appreciable increase in the percent removal as the dosage was increased. This could be due to fewer available active sites than the adsorbates.

As the percent removal of the absorbates increased with an increase in the mass of the adsorbent, the adsorptive capacities decreased. The adsorptive capacity was used as an indicator to select the optimum dosage for the removal of fluoride from the solution. As it could be seen in Fig. 9b, the uptake of fluoride per unit mass decreased with an increase in dosage. As per these results, 70 mg was chosen as the optimum dosage since there was no appreciable increment of percent removal and adsorptive capacity beyond that. This dosage was used for the defluoridation application of real groundwater samples from the study area.

### Isotherm studies

An adsorption isotherm depicts the graphical representation of the adsorptive capacity of an adsorbent against its equilibrium concentrations in the bulk solution at a constant temperature. It gives a general overview of the maximum adsorbates uptake an adsorbent could remove. The two widely used isotherm models; thus, Langmuir and Freundlich were employed in this study to investigate the adsorption process. The nonlinear form of the models as expressed by Eq. (6) and (7) were used. The Langmuir model assumes a monolayer adsorption at a fixed number of well-defined localised sites with no lateral interactions between adsorbates at neighbouring sites. The Freundlich isotherm posits that adsorbate sorption takes place on a heterogeneous surface via multilayer sorption.

**Figure 10:**
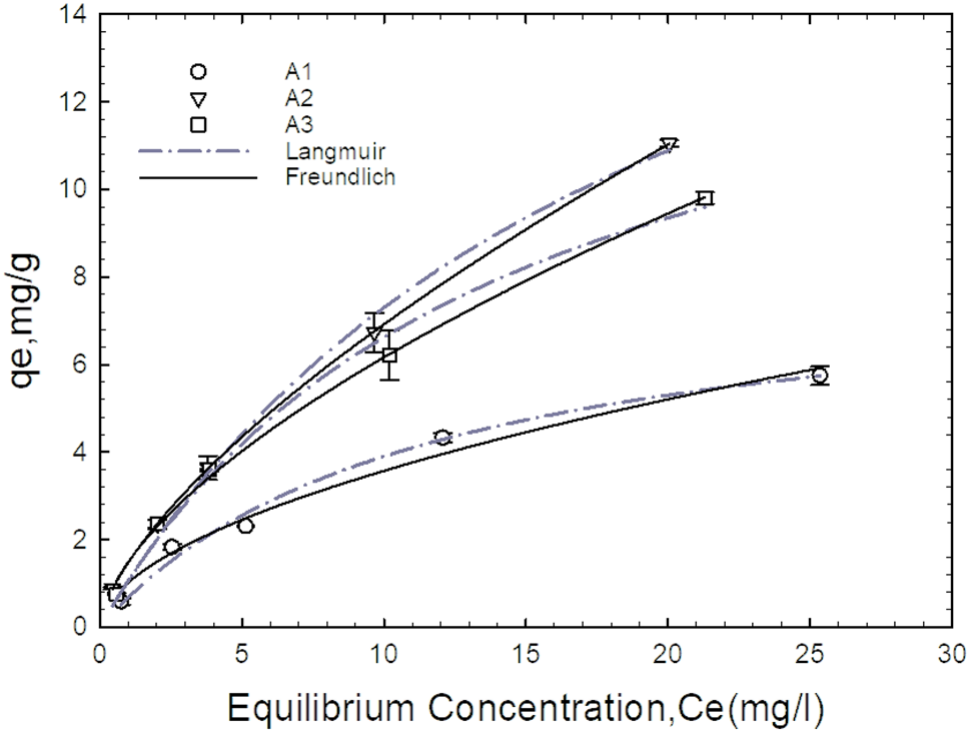
Nonlinear curve fitting of adsorptive capacity as a function of equilibrium concentrations for A1 (as-prepared alumina), A2 (modified alumina with alum ratio 1:0.5), A3 modified alumina with alum ratio 1:1. The error bars represent the standard deviations from the duplicate experiments (NB: some of the error bars are not visible because they are within the size of the marker).

The stronger active sites on the adsorbent are first occupied, and as the sorption process proceeds, the binding energy diminishes. The nonlinear isothermal plot and its associated parameters are given in Fig. 10 and Table 4.

**Table 4:** Results obtained from isotherm curve fitting.

Adsorbents	Langmuir q_m_ (mg/g)	K_L_ (L/mg)	R^2^	Chi-square (X^2^)	Freundlich K_F_ (mg/g)(L/mg)^1/n^	1/n	R^2^	Chi-square (X^2^)
A1	8.271	0.089	0.989	0.0923	1.024	0.543	0.982	0.201
A2	21.404	0.051	0.993	0.249	1.473	0.672	1.000	0.001
A3	15.913	0.071	0.991	0.125	1.491	0.616	0.998	0.134

The coefficient of correlations, R^2^, were too close for both isotherm models to determine which isotherm fits best for the equilibrium data. The chi-square test was additionally used to back up the best-fitting adsorption isotherm model. The best fit model is evaluated by Eq. (11) as;

11 $$x^{2}_{e} = \sum \frac{{\left( {q_{cal} - q_{exp} } \right)^{2} }}{{q_{exp} }}$$

where X_e_^2^ is the chi-square test value, q_cal_ (mg/g) is the calculated adsorptive capacity and q_exp_ (mg/g) is the experimental adsorptive capacity. Therefore, a high correlation coefficient and low chi-square values are used to determine which model fits well for the equilibrium adsorption data. In general, the adsorption of fluoride ions on all the adsorbents has a strong regression coefficient above 0.95.

As it could be inferred from Table 4, data for adsorbent A1 fitted well for Langmuir isotherm than Freundlich isotherm which recorded high R^2^ and low X^2^ values of 0.9895 and 0.0926 respectively. Unlike A1, A2 was more likely to follow the Freundlich isotherm than Langmuir with the highest coefficient of regression and the lowest chi-square value of 1 and 0.001 respectively. Also, A3 had a high value of 0.9975 for the regression coefficient for the Freundlich isotherm than the Langmuir. However, the chi-square value of 0.125 for the Langmuir isotherm was lower than the Freundlich isotherm which makes the adsorption of the fluoride ions onto the A3 adsorbent follow the Langmuir isotherm. The values of 1/n (0.5428, 0.6718, and 0.6164) lying between 0.1 and 1.0 and that of n (1.84, 1.49, and 1.62) lying in the range 1 and 10 for A1, A2, and A3 respectively for both cases demonstrate the strong interaction between the adsorbate and adsorbent as well as the heterogeneous nature of the adsorbent surface. A similar isotherm was obtained by Kumar et al.^49^ in the “defluoridation from aqueous solutions by nano-alumina: Characterization and sorption studies”. Different researchers such as Swain et al.^50^ Tabi et al.^45^ and Zhao et al.^46^ all reported Langmuir isotherm for their fluoride adsorption studies.

The Langmuir maximum adsorptive capacity of the adsorbents in this study was compared with other sorbents reported previously in the literature and the result is presented in Table 5. When compared to some previously developed adsorbents, the A2 adsorbent from this study shows comparable adsorption capacity for defluoridation process. However, in some cases, the adsorbent showed a lower sorption potential.

**Table 5:** Comparison of the effectiveness of different adsorbents for defluoridation process in literature.

Adsorbents	pH	Concentrations (mg/L)	Temperature (◦C)	Adsorptive Capacities (mg/g)	Reference
Alumina modified with alum (w/w 1:0.5)	6.5	1–30	30	21.4	This study
KMnO4 modified carbon	2.0	5–20	25	15.9	^51^
Nano-alumina	6.15	1–100	25	14	^49^
Hydrous-manganese-oxide-coated alumina	5.2	1–70	30	7.09	^52^
Scandinavia spruce wood charcoal (AlFe650/C)	7.0	2–50	28	13.64	^53^
Magnetic-chitosan	7.0	5–140	25	22.49	^54^
Fe3O4@Al (OH)_3_ magnetic nanoparticles	6.5	0–160	25	88.48	^46^
Iron (III)–Tin (IV) mixed oxide	6.4	10–50	30	10.47	^55^
Granular ferric hydroxide (GFH)	6.0–7.0	1–100	25	7.00	^56^
Fe–Al–Ce nano-adsorbent	7.0	2	25	2.22	^57^

### Adsorption kinetic study

The pseudo-first-order (PFO), pseudo-second-order (PSO), and intraparticle diffusion (IP) models were applied to study the kinetics of the adsorption process by the three adsorbents on the different concentrations ranging from 1 to 30 mg/L.

**Figure 11:**
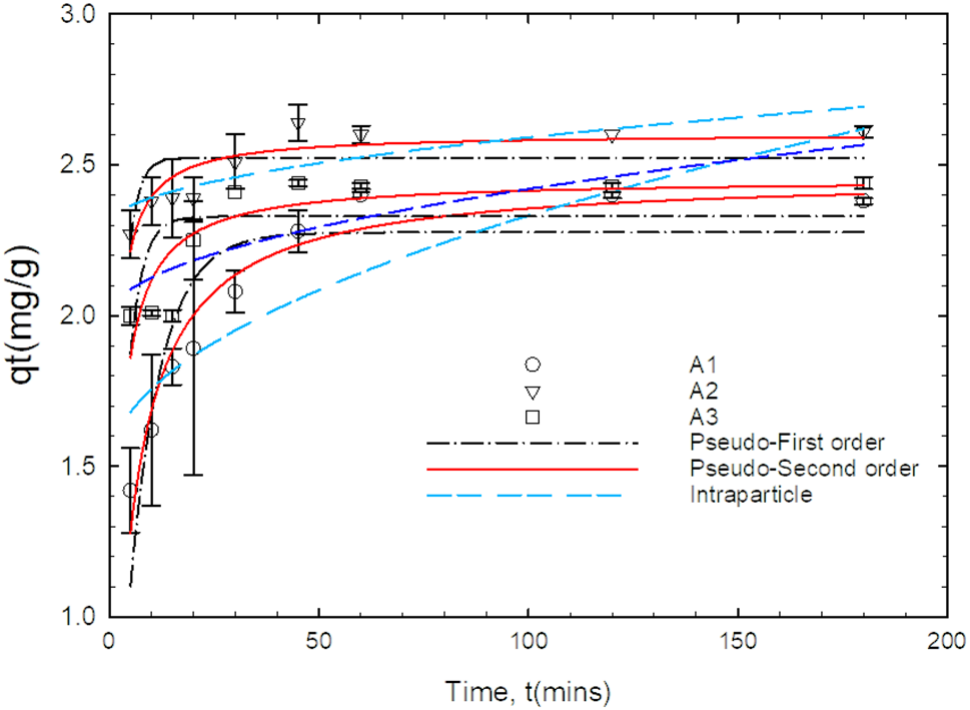
Nonlinear kinetic curve fits of adsorptive capacity as a function of time for the adsorption of fluoride onto A1 (as-prepared alumina), A2 (modified alumina with alum ratio 1:0.5), A3 modified alumina with alum ratio 1:1 at initial fluoride concentration of 5 mg/L, pH 6.5, dosage 10 mg, temp. 30 °C, working volume 10 mL, rotational speed of 150 rpm. The error bars represent the standard deviations from the duplicate experiments (NB: some of the error bars are not visible because they are within the size of the marker).

The PFO, PSO, and intraparticle models are expressed in Eq. (8), (9), and (10) respectively. The nonlinear plot for the initial fluoride concentration of 5 mg/L and its associated parameters are given in Fig. 11 and Table 6. The parameters for the other initial concentrations (1, 10, 15, 30 mg/L) are given in the supplementary information (Sup F4).

**Table 6:** Results obtained from Kinetic studies at initial concentration of 5 mg/L.

Adsorbents	Pseudo-first order, 5 mg/L			
	q_exp_	q_cal_	K_1_	R^2^
A1	2.28	2.2762	0.1326	0.7762
A2	2.64	2.5231	0.4370	0.4301
A3	2.44	2.3295	0.3253	0.3478

Adsorbent	Pseudo-second order			
	qexp	qcal	K_2_	R^2^
A1	2.28	2.4645	0.0876	0.9454
A2	2.64	2.6043	0.4356	0.7993
A3	2.44	2.4527	0.2563	0.7317

Adsorbent	Intraparticle diffusion model			
	C	Kp	R^2^	
A1	1.4905	0.0842	0.7386	
A2	2.2990	0.0293	0.6674	
A3	1.9910	0.0429	0.5952	

At 5 mg/L, A1, A2, and A3 are assumed to follow the PSO kinetics with a higher regression coefficient of 0.9454, 0.7993, and 0.7317 respectively. The calculated adsorptive capacities were found closer to the experimental adsorptive capacities. The various parameters associated with kinetics for the different adsorbents and different concentrations are shown in the supplementary information. Considering the regression coefficient and the consistencies between the calculated and experimental adsorptive capacities, the adsorption of fluoride onto all three adsorbents at the different concentrations follows the pseudo-second-order kinetics. Thus, the adsorption process is more likely to be chemisorption. This could also be inferred from the isotherm studies which were highly influenced by Langmuir isotherm. Studies conducted by these researchers Kumar et al.^49^ Li et al.^58^ and Maliyekkal et al.^59^ found that the uptake of fluoride by the various adsorbents used in their work follows a similar kinetic model.

### Adsorption mechanism

The intraparticle diffusion model as expressed in Eq. (10) was employed to study the plausible mechanism controlling the adsorption process. From the model, it could be inferred that a plot of q_t_ against the square root of time (t) should yield a linear curve if intraparticle diffusion is the rate-determining step. It could be seen that the model would have passed through the origin if the intraparticle (IP) diffusion was the sole determining factor for the adsorption process. The low regression coefficient values obtained suggest that IP may not be the adsorption rate determinant due to a possible smaller size of the fluoride ions as compared to the pores of the adsorbents. The plausible mechanism controlling the fluoride uptake by the adsorbent could either be an electrostatic interaction between the positive charge at the surface of the adsorbent and the negative charge of the fluoride ions or the ion exchange at the surface. This agrees with the previously reported results from literature^60,61^.

### Studies on real groundwater sample

The efficiency of all three adsorbents was evaluated by applying them to fluoride removal of a real groundwater system. Water samples were taken from three communities in the northern part of Ghana namely, Guborigu (TN-B1), Guborigu-Tirigusoka (TN-B2), and Guboriga-yagne (TN-B3). The physico-chemical water parameters from the samples are presented in Table 7.

**Table 7:** Physico-Chemical Parameters of the water samples from the study area.

Parameters	Communities		
	TN-B1	TN-B2	TN-B3
pH	7.9	7.6	7.4
T/°C	18.5	18.6	18.5
EC (uS/cm)	360	440	510
F^-^(mg/L)	1.21	2.5	3.27
SO_4_^2−^ (mg/L)	9	3	4
HCO_3_^-^(mg/L)	148.84	300.12	341.60
PO_4_^2−^ (mg/L)	0.63	0.23	0.13
NO_3_^-^(mg/L)	5.2	1.9	0.9
NO_2_^-^(mg/L)	0.012	0.022	0.01
Total hardness (mg/L)	110	215	170
Ca hardness(mg/L)	20.04	13.63	9.62
Mg hardness(mg/L)	89.96	180.93	145.95
Mg^2+^ (mg/L)	21.86	43.98	35.47
Ca^2+^ (mg/L)	8.02	13.63	9.62
Turbidity (NTU)	0.29	0.29	0.26
Cl^-^(mg/L)	29.99	244.92	109.97

**Figure 12:**
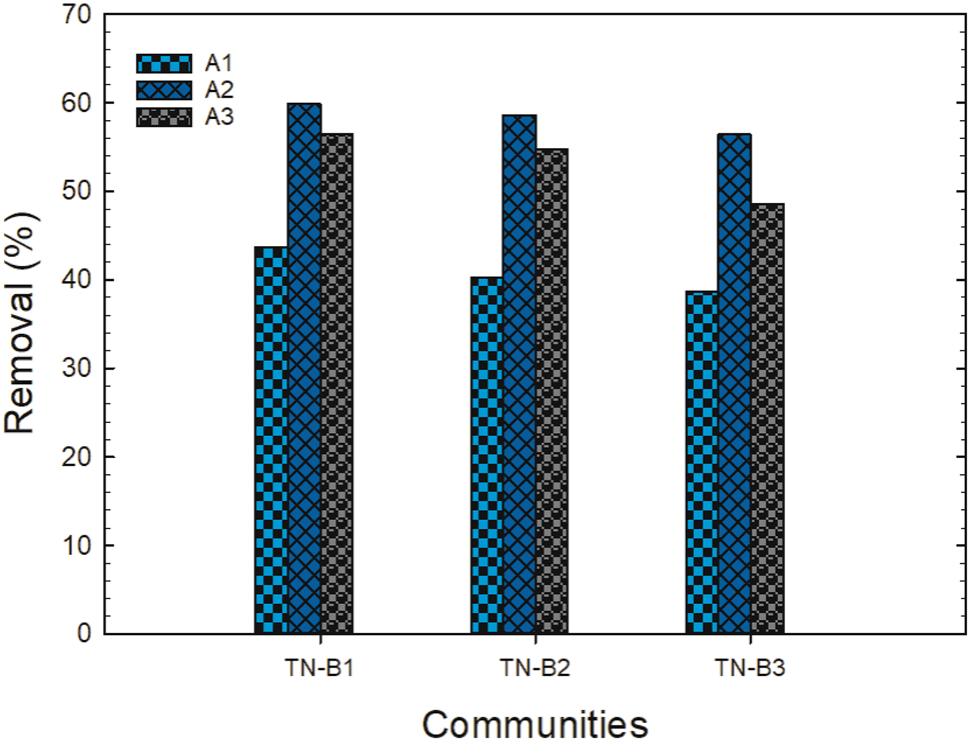
Percentage fluoride removal from groundwater at time 1 h, dosage 70 mg, working volume 10 mL, rotational speed of 150 rpm for A1 (as-prepared alumina), A2 (modified alumina with alum ratio 1:0.5), A3 (modified alumina with alum ratio 1:1).

The removal of fluoride from real drinking groundwater was done at the optimum experimental conditions of adsorbent dosage;70 mg, working volume of 10 mL; equilibrium contact time of 1 h, and a rotational speed of 150 rpm. The results are shown in Fig. 12.

The percent removal of fluoride from the groundwater at the optimum conditions is lower, as compared to what is seen in Fig. 9. There are a whole lot of ions that are present in groundwater systems that might vie with the adsorption of fluoride from the water. The highest fluoride uptake was recorded in TN-B1 by A2 with an initial fluoride concentration of 1.21 mg/L. The difference in the percent removal at TN-B1 and TN-B2 by A2 was not much significant, meanwhile TN-B2 had a higher fluoride concentration of 2.5 mg/L and that of TN-B1 is 1.21 mg/L. The plausible explanation for this observation is that the concentrations of SO_4_^2−^, PO_4_^2^ and NO_3_^-^ measured from TN-B1 were higher than TN-B2. However, the concentrations of HCO_3_^-^ and Cl^-^ for TN-B1 were lower than TN-B2. This suggests that the multivalent anions are more readily adsorbed than monovalent anions and therefore the uptake of fluoride ions by the adsorbents is hindered by these anions as they compete with fluorides on the same available active sites during the sorption process.

## Conclusion

Alumina obtained from aluminium foil with modified alum was found to be an efficient adsorbent for the removal of fluoride from simulated water and real groundwater. The maximum percent removal obtained at the optimum conditions of 1 h, 70 mg adsorbent dosage, working volume of 10 mL, pH of 6.0, a temperature of 30 °C, and shaking speed of 150 rpm was 96%. The static experiment conditions were used to calibrate the PHREEQC geochemical model which was used to simulate the adsorption of fluoride onto the modified alumina at different conditions. PHREEQC was also coupled with parameter estimation software (PEST) to determine equilibrium constants for the surface reactions between the fluoride species and the adsorbent in a way that the simulations accurately reflect the outcomes of laboratory experiments. According to the study, it is possible to precisely predict how fluoride would react with the adsorbent under various conditions when PHREEQC and PEST are combined. Therefore, before carrying out the sorption process, one can utilize PHREEQC modelling and the findings from this study to predict the amount of the adsorbent to be employed based on feed water quality and the volume of the water to be treated. The successful integration of PHREEQC with PEST demonstrates that predictions of fluoride sorption processes onto modified alumina are possible using sparse experimental data.

The isotherm studies suggest that Langmuir isotherm fitted very well for the equilibrium data obtained from all the different adsorbents used except A2, with a high regression coefficient and low chi-square values which indicates monolayer sorption on the homogeneous adsorbent surface.

Pseudo-first order and Pseudo-second order models were employed on the sorption data obtained to study the sorption kinetics. The data fitted better to the pseudo-second-order which further indicate that the sorption process was influenced by chemical interactions between the adsorbates and the adsorbent. The mechanism for the sorption of the fluoride ions was studied by the intraparticle (IP) diffusion model which revealed that IP was not the rate-determining factor, and therefore the most plausible mechanism controlling the fluoride uptake by the adsorbent could either be an electrostatic interaction between the positive charge at the surface of the adsorbent and the negative charge of the fluoride ions or the ion exchange at the surface.

The findings obtained from this research show that readily available waste could be valorised into a useful product that could be employed in the removal of fluoride from water samples, including groundwater, that may contain too much fluoride and pose a risk to the general public's health.

## Supplementary Information

Supplementary Information 1.

Supplementary Information 2.

Supplementary Information 3.

Supplementary Information 4.

## Data Availability

The data that support the findings of this study are provided in the manuscript.

## Code availability

All relevant codes used for the modelling, simulation and optimization are available in the manuscript and also in the supplementary files.
