# Supplementary material for: Understanding fluoride adsorption from groundwater by alumina modified with alum using PHREEQC surface complexation model
Source: Sci Rep. 2023 Jul 29;13:12307. doi: 10.1038/s41598-023-38564-1 (PMC10387067; doi:10.1038/s41598-023-38564-1)
Supplement: Supplementary file 4 — Supplementary Information 4. [file 41598_2023_38564_MOESM4_ESM.docx]

**Supplementary information**

**Understanding fluoride adsorption from groundwater by alumina modified with alum using PHREEQC surface complexation model**

Francis Adu-Boahene^1^; Patrick Boakye^2,4^*; Frank Ofori Agyemang^3^; Jolly Kanjua^4^, Sampson Oduro‑Kwarteng^1^

^1^ *Department of Civil Engineering (Regional Water and Environmental Sanitation Centre, Kumasi), Kwame Nkrumah University of Science and Technology, PMB, Kumasi, Ghana*.

^2^ *Department of Chemical Engineering, Kwame Nkrumah University of Science and Technology, PMB, Kumasi, Ghana.*

*^3^ Department of Materials Engineering, Kwame Nkrumah University of Science and Technology, PMB, Kumasi, Ghana*.

^4^ *Institute of Computation and Neuroscience, Apemso, Kumasi, Ghana.*

* Corresponding email: [patrickboakye@knust.edu.gh](mailto:patrickboakye@knust.edu.gh)

1. Kinetic studies

**Table S 1. Results obtained from Kinetic studies at initial concentration of 1 mg/L**

| Pseudo-First Order, 1 mg/L | | | | |
| --- | --- | --- | --- | --- |
| Adsorbents | $\boldsymbol{q}_{\boldsymbol{exp}}$ | $\boldsymbol{q}_{\boldsymbol{cal}}$ | **K_1_** | **R^2^** |
| A1 | 0.58 | 0.5725 | 0.0322 | 0.9377 |
| A2 | 0.86 | 0.8147 | 0.3675 | 0.3799 |
| A3 | 0.74 | 0.7042 | 0.1985 | 0.5388 |
|  |  |  |  |  |
| Pseudo- Second Order | | | | |
| Adsorbent | $\boldsymbol{q}_{\boldsymbol{exp}}$ | $\boldsymbol{q}_{\boldsymbol{cal}}$ | **K_2_** | **R^2^** |
| A1 | **0.58** | **0.6998** | **0.0463** | **0.8967** |
| A2 | **0.86** | **0.8514** | **0.9089** | **0.7530** |
| A3 | **0.74** | **0.7520** | **2.8281** | **0.8408** |
|  |  |  |  |  |
| Intraparticle Diffusion Model | | | | |
| Adsorbent | **C** | $\boldsymbol{K}_{\boldsymbol{p}}$ | **R^2^** |  |
| A1 | **0.0584** | **0.0445** | **0.7444** |  |
| A2 | **0.7141** | **0.0130** | **0.6307** |  |
| A3 | **0.5358** | **0.0194** | **0.6816** |  |
|  |  |  |  |  |

**Table S 2. Results obtained from Kinetic studies at initial concentration of 10 mg/L**

| Pseudo-First Order, 10 mg/L | | | | |
| --- | --- | --- | --- | --- |
| Adsorbents | $\boldsymbol{q}_{\boldsymbol{exp}}$ | $\boldsymbol{q}_{\boldsymbol{cal}}$ | **K_1_** | **R^2^** |
| A1 | **4.05** | **4.0237** | **0.1040** | **0.9066** |
| A2 | **4.30** | **4.1474** | **0.4775** | **0.3171** |
| A3 | **4.20** | **4.0187** | **0.3830** | **0.4621** |
|  |  |  |  |  |
| Pseudo- Second Order | | | | |
| Adsorbent | $\boldsymbol{q}_{\boldsymbol{exp}}$ | $\boldsymbol{q}_{\boldsymbol{cal}}$ | **K_2_** | **R^2^** |
| A1 | **4.05** | **4.4610** | **0.0321** | **0.8587** |
| A2 | **4.30** | **4.2746** | **0.2952** | **0.7565** |
| A3 | **4.20** | **4.1818** | **0.2061** | **0.8205** |
|  |  |  |  |  |
| Intraparticle Diffusion Model | | | | |
| Adsorbent | **C** | $\boldsymbol{K}_{\boldsymbol{p}}$ | **R^2^** |  |
| A1 | **2.2576** | **0.1783** | **0.4844** |  |
| A2 | **3.8029** | **0.0462** | **0.7091** |  |
| A3 | **3.5544** | **0.0601** | **0.7165** |  |
|  |  |  |  |  |

**Table S 3. Results obtained from Kinetic studies at initial concentration of 15 mg/L**

| Pseudo-First Order, 15 mg/L | | | | |
| --- | --- | --- | --- | --- |
| Adsorbents | $\boldsymbol{q}_{\boldsymbol{exp}}$ | $\boldsymbol{q}_{\boldsymbol{cal}}$ | **K_1_** | **R^2^** |
| A1 | **3.25** | **3.1107** | **0.2347** | **0.7263** |
| A2 | **8.23** | **7.6052** | **0.2237** | **0.3507** |
| A3 | **6.69** | **6.3432** | **0.1656** | **0.5005** |
|  |  |  |  |  |
| Pseudo- Second Order | | | | |
| Adsorbent | $\boldsymbol{q}_{\boldsymbol{exp}}$ | $\boldsymbol{q}_{\boldsymbol{cal}}$ | **K_2_** | **R^2^** |
| A1 | **3.25** | **3.2894** | **0.1371** | **0.9370** |
| A2 | **8.23** | **8.2014** | **0.0435** | **0.7265** |
| A3 | **6.69** | **6.7757** | **0.0442** | **0.7614** |
|  |  |  |  |  |
| Intraparticle Diffusion Model | | | | |
| Adsorbent | **C** | $\boldsymbol{K}_{\boldsymbol{p}}$ | **R^2^** |  |
| A1 | **2.5234** | **0.0679** | **0.6224** |  |
| A2 | **5.7423** | **0.2289** | **0.7283** |  |
| A3 | **4.6159** | **0.1907** | **0.6087** |  |
|  |  |  |  |  |

**Table S 4. Results obtained from Kinetic studies at initial concentration of 30 mg/L**

| Pseudo-First Order, 30 mg/L | | | | |
| --- | --- | --- | --- | --- |
| Adsorbents | $\boldsymbol{q}_{\boldsymbol{exp}}$ | $\boldsymbol{q}_{\boldsymbol{cal}}$ | **K_1_** | **R^2^** |
| A1 | **5.95** | **5.4483** | **0.1277** | **0.5319** |
| A2 | **7.85** | **7.6076** | **0.0858** | **0.9447** |
| A3 | **7.05** | **7.1844** | **0.1655** | **0.9280** |
|  |  |  |  |  |
| Pseudo- Second Order | | | | |
| Adsorbent | $\boldsymbol{q}_{\boldsymbol{exp}}$ | $\boldsymbol{q}_{\boldsymbol{cal}}$ | **K_2_** | **R^2^** |
| A1 | **5.95** | **5.8358** | **0.0977** | **0.7494** |
| A2 | **7.85** | **8.3617** | **0.06156** | **0.9414** |
| A3 | **7.05** | **7.7324** | **0.0878** | **0.9371** |
|  |  |  |  |  |
| Intraparticle Diffusion Model | | | | |
| Adsorbent | **C** | $\boldsymbol{K}_{\boldsymbol{p}}$ | **R^2^** |  |
| A1 | **3.6154** | **0.1925** | **0.6271** |  |
| A2 | **4.0141** | **0.3484** | **0.6186** |  |
| A3 | **5.1458** | **0.2229** | **0.5904** |  |
|  |  |  |  |  |
